# Characterization of c-Maf^+^Foxp3^−^ Regulatory T Cells Induced by Repeated Stimulation of Antigen-Presenting B Cells

**DOI:** 10.1038/srep46348

**Published:** 2017-04-12

**Authors:** Chien-Hui Chien, Hui-Chieh Yu, Szu-Ying Chen, Bor-Luen Chiang

**Affiliations:** 1 Graduate Institute of Clinical Medicine, National Taiwan University, Taipei City, 10048, Taiwan R.O.C; 2 Department of Medical Research, National Taiwan University Hospital, Taipei City, 10002, Taiwan R.O.C

## Abstract

The role of B cells in the development of CD4^+^ regulatory T cells has been emphasized recently. Our previous studies have demonstrated that the antigen-presenting splenic B cells converted naïve CD4^+^CD25^−^ T cells into CD4^+^CD25^+^Foxp3^−^ T cells without additional cytokines or chemicals with regulatory activity and that referred to as Treg-of-B cells. The present study further showed that Treg-of-B cells increased the IL-10-producing population, and the expression of c-Maf, inducible T-cell co-stimulator (ICOS) as well as cytotoxic T-lymphocyte-associated protein 4 (CTLA4) after repeated stimulation of B cells in a cell-cell contact-dependent manner. Long-term cultured Treg-of-B cells exerted IL-10 and CTLA4-mediated antigen-specific suppressive activity; moreover, the single antigen-specific Treg-of-B cells inhibited in a non-antigen-specific fashion. In conclusion, these results suggest that repeated stimulation of B cells induced IL-10-producing CD4^+^Foxp3^−^ regulatory T cells in a contact-dependent manner and these Treg-of-B cells possess IL-10 and CTLA4-dependent suppressive function.

The role of B cell as antigen-presenting cell (APC) for CD4^+^ T cell response is not well defined. Reports suggest that B cells are involved in the tolerance response to self-antigens derived from the anterior chamber of the eye^1^,^2^,^3^ and thymus^4^,^5^,^6^ as well as foreign antigens from the mucosal route^7^,^8^,^9^. Adoptive transfer of B cells prevented the T cell-induced inflammatory bowel disease and also the chemical-induced colitis^9^,^10^,^11^. The antigen-presenting B cells induced antigen-specific CD4^+^ T cells proliferation *in vivo* and exhibited a decreased proliferative response to antigen *ex vivo* with a controversial observation of forkhead box P3 (Foxp3) expression^12^,^13^. Furthermore, *in vitro* naïve B cells had an ability to convert naïve CD4^+^ T cells into Foxp3^−^CD62L^hi^CD25^+^ Treg cells without additional cytokines through mature immunological synapses but not by bone marrow-derived dendritic cells^14^. Other studies showed that B cells enhanced the induction of Foxp3^+^ Treg cells in the presence of transforming growth factor (TGF)-β and interleukin (IL)-2^15^ and B cells preferentially induced Foxp3^+^ Treg cells proliferation in an antigen-specific manner^16^. These studies highlight the role of B cells in the induction of tolerance of CD4^+^ T cells although the underlying mechanisms remain unclear.

The most well-known inducible Foxp3^−^ Treg cells is type 1 regulatory (Tr1) cells that can be induced by additional IL-10^17^,^18^ or IL-27^19^,^20^,^21^ in the culture. By microarray analysis, IL-10-induced human Tr1 cell clone expressed CD49b, LAG3, and CD226 and similar expression pattern also observed in murine IL-10-producing T cells^18^. Another *in vivo* study demonstrated that IL-10-producing CD4^+^ T cells expressed higher levels of c-Maf and IL-21 after intranasal anti-CD3ε antibody treatment and these expressions also observed on the IL-27-driven Tr1 cells *in vitro*^20^. T cell activation in the presence of vitamin D3 and dexamethasone induced the IL-10-producing T cells with regulatory function but not Foxp3 expression^22^,^23^. In addition to soluble factors, the inducible T-cell co-stimulator (ICOS)-ICOS ligand and glucocorticoid-induced TNFR-related (GITR)-GITR ligand pathways also reported to being critical for IL-10-producing T cells generation^19^,^24^,^25^,^26^,^27^.

Our previous studies reported that splenic B cells have the potential to induce CD4^+^CD25^+^Foxp3^−^ Treg cells, which named as Treg-of-B cells, with suppressive function as well as the Peyer’s patches B cells and peritoneal B-1a cells^28^,^29^,^30^,^31^,^32^. Treg-of-B cells have been demonstrated that protected mice from allergic asthma, arthritis, and inflammatory bowel disease^28^,^30^,^31^,^32^,^33^. Peptide-induced antigen-specific Treg-of-B cells and monoclonal antibodies induced non-antigen-specific Treg-of-B cells possessed suppressive function. Furthermore, antigen-specific Treg-of-B cells exerted non-antigen-specific suppressive function both *in vitro* and *in vivo*^31^. The present study investigated that whether repeated stimulation of B cells induced Treg-of-B cells with enhanced regulatory ability or effector function. Long-term cultured with B cells promoted the generation of Treg-of-B cells with expressions of ICOS, PD1, LAG3, CTLA4, c-Maf, and IL-10 in a cell-cell contact fashion rather than soluble factors or cytokines. The present results suggested that repeated stimulation of B cell induced Foxp3^−^ Treg-of-B cells with IL-10 and CTLA4-dependent suppressive functions.

## Results

### Repeated stimulation of B cell-induced CD4^+^ IL-10-producing Treg cells

**Figure 1:**
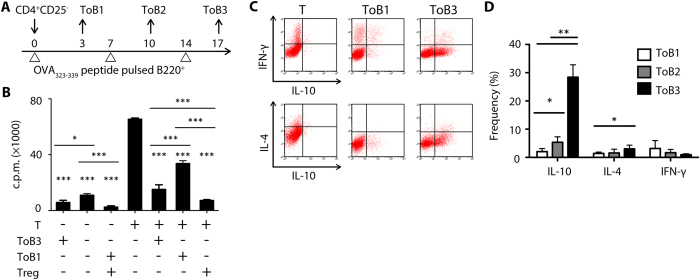
B cells induced IL-10-producing T cells without additional IL-10. (**A**) CD4^+^CD25^−^ T cells cultured with OVA_323–339_ peptide-pulsed B cells (Day 0, 7, 14) for 3 days and then cells rested in medium supplement with recombinant IL-2 (Day 3 and 10) for 4 days. Treg-of-B (ToB)-1, -2, and -3 cells were collected by depleting B cells. (**B**) The suppressive assay. Splenic CD4^+^CD25^−^ (T) cells seeded as responder cells with suppressor cells, including Treg-of-B cells and splenic CD4^+^CD25^+^ (Treg) cells, at a ratio of 1:1. Data shown were the representative of 3 individual experiments. (**C**) Representative intracellular cytokine staining in splenic naïve CD4^+^CD25^−^ (T) and Treg-of-B cells. (**D**) The cumulative data of frequency of IL-10-, IFN-γ- and IL-4-producing populations in reactivated Treg-of-B cells from 4–6 individual experiments.

The present study investigated the effect of B cells as APCs on naïve CD4^+^CD25^−^ T cells without additional cytokine supplements. T cells collected at different co-culture rounds with OVA_323–339_ peptide-pulsed B cells as Treg-of-B (ToB)-1, 2, and -3 (Fig. 1A). After repeated stimulation of B cells, OVA-specific Treg-of-B3 cells enhanced anergic response and suppressive function in antigen-specific (Fig. 1B) and non-antigen-specific manners (see Supplementary Fig. S1A). Treg-of-B3 cells also increased the frequency of IL-10- and IL-4-producing populations but decreased the frequency of interferon (IFN)-γ-producing population (Fig. 1C,D). These results indicated that repeated stimulation of antigen-presenting B cells, without additional IL-10 or other cytokine supplements, converted CD4^+^CD25^−^ T cells into IL-10-producing Treg-of-B cells.

### IL-10-producing Treg-of-B cells expressed c-Maf not Foxp3

**Figure 2:**
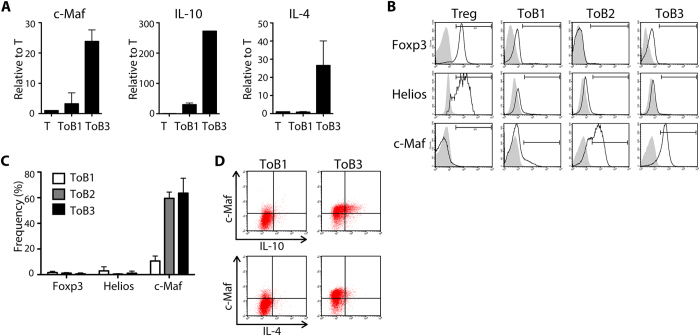
B cell-induced IL-10-producing T cells expressed c-Maf but not Foxp3. (**A**) The mRNA levels of c-Maf, IL-10, and IL-4 in Treg-of-B cells compared to that of splenic CD4^+^CD25^−^ (T) cells. (**B**) Representative histogram data of Foxp3, Helios, and c-Maf in Treg-of-B cells and CD4^+^CD25^+^ (Treg) cells. (**C**) The cumulative data of frequency of Foxp3, Helios, and c-Maf-expressing populations in Treg-of-B cells from 6 individual experiments. (**D**) Representative flow plots of co-expression of c-Maf, IL-10, and IL-4 in Treg-of-B cells from 3 individual experiments.

Next, we analyzed the expression of transcriptional factors in Treg-of-B cells. The expression level of c-Maf increased significantly after multiple B-T co-cultures among the well-known transcriptional factors that regulated IL-10 promoter region in our microarray results (data not shown). In consistent with these, the expression of c-Maf increased in Treg-of-B3 cells compared to that of Treg-of-B1 cells in both mRNA and protein levels (Fig. 2A–C). Treg-of-B cells increased the expression of c-Maf after multiple co-cultures but did not alter the expression of Helios or Foxp3 as naïve CD4^+^CD25^+^ (Treg) cells did (Fig. 2B,C). We found that the mRNA levels of IL-10 and IL-4 elevated in Treg-of-B3 cells; furthermore, the major IL-10- and IL-4-producing Treg-of-B3 cells confined to the c-Maf-expressing populations (Fig. 2A,D). These results suggested that repeated stimulation of antigen-presenting B cells induced IL-10 expression in Treg-of-B cells might be via the activation of c-Maf.

### Treg-of-B cells differed from the IL-10-induced Tr1 cells

**Figure 3:**
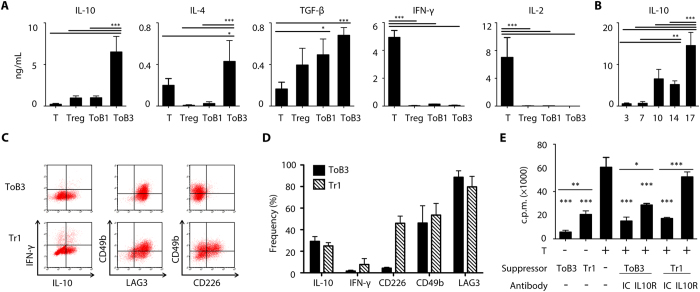
B cell-induced IL-10-producing T cells differed from the IL-10-induced Tr1 cells. (**A**) Cytokines production of reactivated splenic CD4^+^CD25^−^ (T), CD4^+^CD25^+^ (Treg), Treg-of-B1 (ToB1), and Treg-of-B3 (ToB3) cells. (**B**) The cumulative data of IL-10 level of Treg-of-B cells at cultured day 3, 7, 10, 14, 17 from 9 individual experiments. (**C**) Representative flow plots of markers on Treg-of-B3 cells and IL-10-induced Tr1 cells from 3 individual experiments. (**D**) The cumulative data of frequency of marker-expressing populations in Treg-of-B cells from 3 individual experiments. (**E**) Suppression assay in the presence of 10 μg mL^−1^ anti-IL-10 receptor (IL10R) or isotype control (IC) antibodies with or without transwell insertion. Data shown were the representative of 3 individual experiments.

The cytokine profiles of activated Treg-of-B cells were analyzed and compared to the primary purified splenic CD4^+^CD25^−^ (T) and CD4^+^CD25^+^ T (Treg) cells. After multiple co-cultures, Treg-of-B3 cells increased the production of IL-10 and IL-4 and decreased the production of IFN-γ and IL-2 compared to that of Treg-of-B1 cells (Fig. 3A). The level of IL-10 elevated significantly during Treg-of-B3 induction (Fig. 3B), and these raised a question that the difference between Treg-of-B cells and the IL-10-induced Tr1 cells. We cultured Treg-of-B cells and IL-10-driven Tr1 cells simultaneously to compare their marker expression as well as suppressive functions. Treg-of-B3 cells had less IFN-γ-producing or CD226^+^ populations; although, Treg-of-B3 cells and Tr1 cells had the similar frequency in IL-10-producing, LAG3^+^, and CD49b^+^ populations (Fig. 3C,D). In addition, IL-10 played a role but not the only role in the suppressive function of Treg-of-B3 cells whereas the suppressive function of Tr1 cells relied on the IL-10 secretion (Fig. 3E). IL-10-medieated inhibition was also involved in Treg-of-B3 cells by non-antigen-specific suppressive mechanism (see Supplementary Fig. S1B). These results suggested that Treg-of-B cells differed from the IL-10-induced Tr1 cells in phenotype and suppressive ability.

### Treg-of-B cells suppression through IL-10 and CTLA4

**Figure 4:**
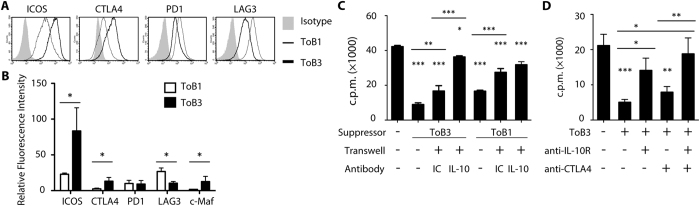
IL-10 and CTLA4 played roles in the suppressive function of Treg-of-B cells. (**A**) The representative of levels of ICOS, CTLA4, PD1 and LAG3 on Treg-of-B1 (ToB1; thin) and Treg-of-B1 (ToB1; thick) cells. Isotype control (filled). Data shown were the representative of at least 3 individual experiments. (**B**) The cumulative results of relative fluorescence intensity of ICOS, CTLA4, PD1, LAG3, and c-Maf on Treg-of-B cells from 3–4 individual experiments. (**C**) IL-10 neutralization assay with the presence of transwell or not. Data shown were the representative of 3 individual experiments. (**D**) Suppression assay with the presence of 10 μg mL^−1^ anti-IL-10 receptor (anti-IL-10R) and anti-CTLA4 antibodies or not. Data shown were the representative of 4 individual experiments.

Next, the phenotype of Treg-of-B cells with multiple B-T co-cultures was analyzed. Treg-of-B cells with multiple co-cultures increased the expression levels of ICOS, CTLA4, and c-Maf whereas decreased the expression level of LAG3 (Fig. 4A,B). The suppressive assay was performed with the insertion of transwell to exclude the cell-cell contact dependent suppression. Strikingly, Treg-of-B3 cells less relied on the cell contact-dependent suppression as Treg-of-B1 did and IL-10 involved in the soluble factor-dependent suppression of Treg-of-B3 cells (Fig. 4C). We further investigated the effect of increased expressions of CTLA4 and IL-10 of Treg-of-B3 cells compared to that of Treg-of-B1 cells. The blockade of IL-10 receptor reversed the suppression of Treg-of-B3 cells and the combination with the blockade of CTLA4 enhanced the reversion (Fig. 4D). The similar results also demonstrated in the CFSE-labeled suppressive assay, Treg-of-B3 cells inhibited responder T cells proliferation in a CTLA4 and IL-10-dependent manner rather than LAG3 (see Supplementary Fig. S4). Furthermore, the present study investigated the effect of repeated stimulation of B cell in a non-antigen-specific manner in the presence of anti-CD3 and anti-CD28 monoclonal antibodies (see Supplementary Fig. S2). Non-antigen-specific Treg-of-B3 cells shared characteristics with antigen-specific Treg-of-B3 cells, including the expressions of ICOS, CTLA4, PD1, LAG3, c-Maf, and IL-10, and anergic phenotype as well as the suppressive activity through IL-10 and CTLA4. These results suggested that IL-10 and CTLA4 played important roles in the suppressive ability of Treg-of-B cells with multiple B-T co-cultures.

### B cell-induced regulatory T cells in a contact-dependent manner

**Figure 5:**
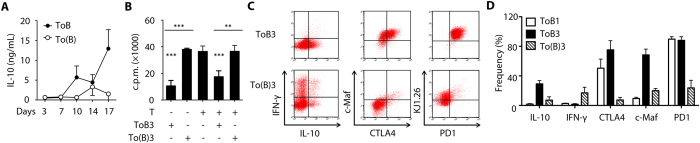
B cells induced Treg cells in a contact-dependent manner. (**A**) The cumulative data of IL-10 level of Treg-of-B cells or T-of-B cells with transwell, [To(B)] during the culture period from 3 individual experiments. (**B**) Suppression assay. (**C**) Representative flow plots of markers on ToB3 and To(B)3 cells from 2 individual experiments. (**D**) The cumulative data of frequency of marker-expressing populations in ToB1, ToB3, and To(B)3 cells from 2–3 individual experiments. Data shown were the representative of at least 3 individual experiments.

We investigated the inductive mechanism of Treg-of-B cells. The levels of IL-10, IL-27, and TGF-β increased during Treg-of-B3 cells induction (Fig. 3B and see Supplementary Fig. S3A). However, IL-10-derived from B cell did not play a role in the induction of suppressive Treg-of-B3 cells (see Supplementary Fig. S3B). In consistent with these, the neutralization of IL-10 and IL-27 plus TGF-β during Treg-of-B3 cells culture period did not alter their suppressive function (see Supplementary Fig. S3C). Then, the role of cell-cell contact during B-T co-cultures was determined by insertion of transwell. As the presence of transwell, T-of-(B) [To(B) in the figure] cells did not increase the level of IL-10 in culture circumstances (Fig. 5A) and T-of-(B) cells did not possess anergic phenotype or suppressive function significantly (Fig. 5B). We found that T-of-(B) cells expressed lower levels of c-Maf, CTLA4, IL-10, PD1, and OVA-specific transgenic TCR (Fig. 5C,D). These results indicated that cell-cell contact was required for the induction of IL-10-producing Treg-of-B cells.

### IL-10-producing regulatory T cells induced by repeated stimulation of B cells

**Figure 6:**
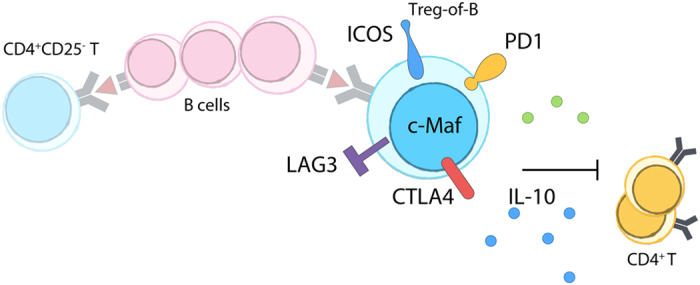
Treg-of-B cells expressed c-Maf and suppressed through CTLA4 and IL-10. Repeated stimulation of antigen-presenting B cells converted naïve CD4^+^CD25^−^ T cells into Foxp3^−^CD4^+^CD25^+^ T cells with regulatory function. After long-term B-T cell-cell contact, Treg-of-B cells enhanced expressions of c-Maf, ICOS, CTLA4, and the production of IL-10. Treg-of-B cells exerted suppressive functions through CTLA4 and IL-10.

Repeated stimulation of B cells converted naïve CD4^+^CD25^−^ T cells into c-Maf-expressing IL-10-producing CD4^+^CD25^+^ Treg-of-B cells in a cell-cell contact fashion (Fig. 6). The long-term B cell stimulation induced Treg-of-B cells expressed c-Maf, ICOS, PD1, LAG3, CTLA4, IL-10, IL-4, and TGF-β. The expression of ICOS, CTLA4, and IL-10 increased after multiple B-T co-cultures; moreover, IL-10 and CTLA4 played the important roles in their regulatory activity. The present study demonstrated a new IL-10-producing Foxp3^−^ Treg cell-type that induced in a contact-dependent manner with B cells and possessed suppressive activity through IL-10 and CTLA4.

## Discussion

It has been known that B cells play the immunomodulatory role physiologically^34^,^35^. Recent studies suggest that B cell participated in the tumor-associated regulatory immune response^36^,^37^. Hitherto, we have reported the ability of B cells derived from spleen, Peyer’s patches, and peritoneal cavity had the potential to induce CD4^+^CD25^+^Foxp3^−^ Treg cells *in vitro*^28^,^29^,^30^,^31^,^32^. Treg-of-B cells expressed CD25, ICOS, PD1, and LAG3 and this pattern conserved in strains and stimulations as well as the origin of B cells. Treg-of-B cells protected mice from Th2 cells mediated airway hyperresponsiveness, airway inflammation, and hyper-produced IgE in allergic asthma in both antigen-specific and antigen-specific fashion^28^,^30^,^31^. Non-antigen-specific Treg-of-B cells induced by anti-CD3 and anti-CD28 monoclonal antibodies prevented mice from osteolysis and joint inflammation in collagen-induced arthritis^32^; furthermore, Treg-of-B cells protected mice from T-cell-induced colitis in an IL-10-independent manner^33^. In this study, we reported that Treg-of-B cells with repeated stimulation of antigen-presenting B cells increased the expression of IL-10, c-Maf, ICOS, and CTLA4 and the suppressive function through IL-10 and CTLA4. The non-antigen-specific Treg-of-B cells induced by monoclonal antibodies shared similar phenotype and the IL-10 and CTLA4-mediated regulatory activities. Furthermore, the single antigen-specific Treg-of-B cells increased their inhibitory ability in a non-antigen-dependent manner. Our data also suggested that cell-cell contact rather than IL-10 or IL-27 played the crucial role of Treg-of-B cell generation. In conclusion, these results indicated repeated stimulation of B cells induced CD4^+^Foxp3^−^ T cells acquired suppressive ability through IL-10 and CTLA4 in the cell-cell contact dependent manner.

CD4^+^Foxp3^−^ Treg-of-B cells proliferated during B-T co-cultures but hypoproliferated during the suppressive assay. This observation was consistent with that self-antigen-bearing B cells stimulated antigen-specific T cells proliferation *in vivo*^13^,^38^. B-cells-activated self-antigen-specific CD4^+^ T cells decreased proliferative capacity *ex vivo*^12^,^39^,^40^, and which unresponsiveness without association with Foxp3 expression^13^. Addition to self-antigens, genetically B cell-deficient mice had a defected tolerogenic response to antigen from the oral route or nasal route^8^,^41^. In contrary to these, the frequency of intrathymic B cells correlated with thymic Foxp3^+^ Treg cells suggesting that thymic B cells promote the development and generation of thymic Foxp3^+^ Treg cells in an antigen-MHCII and CD40/CD80/CD86-dependent manner^4^,^5^,^6^. In summary, B cells play the roles in the induction of CD4^+^ regulatory T cells yet the underlying mechanisms still largely unknown.

The present study showed that long-term cultured Treg-of-B cells expressed LAG3 and CD49b similar to IL-10-induced Tr1 cells; however, Treg-of-B cells did not express CD226 or IFN-γ as the Tr1 cells did. Treg-of-B cells possessed contact-dependent and contact-independent suppressive mechanisms via CTLA4 and IL-10 and that differentiated Treg-of-B cells from the well-known iTreg cells, including Tr1, Th3, and Foxp3^+^ Treg cells. Tr1 cells are characterized by the high amount of IL-10 production with the less amount of IFN- γ and the suppression majorly depend on IL-10^17^,^42^,^43^. Human IL-10-induced Tr1 cell clones expressed CD49b, LAG3, and CD226^18^ and CD226 involved in the activation of human Tr1 cells-mediated lysis of myeloid APCs^44^. Although there was no study reported that murine Tr1 cells lysis APCs via CD226, we supposed that the CD226-mediated Tr1 cell lysis mechanism would be clearly detected in human Tr1 cell clones rather the murine enriched Tr1 cell population. Bacchetta *et al*. showed that the up-regulation of CTLA4 on antibodies activated human IL-10-induced Tr1 cell clones yet demonstrated that CTLA4 play a role in the suppressive function of Tr1 cells^45^. In addition to the suppressive mechanism, the induction of Treg-of-B cells did not require additional cytokine supplement that differed from IL-10-driven Tr1 cells. These above characteristics distinguished Treg-of-B cells into a new type of inducible regulatory T cells.

IL-10-producing Treg-of-B cells were confined to the c-Maf-expressing population, and T-of-(B)3 cells decreased IL-10 and c-Maf-expressing populations. These suggest that repeated stimulation of antigen-presenting B cells induced IL-10-producing Treg-of-B cells possibly by the activation of c-Maf. In fact, c-Maf cooperated with several transcriptional factors and regulated expression of IL-4, IL-10, and IL-21 and in different CD4^+^ T helper (Th) cells, CD8^+^ T cells, and macrophages^21^,^46^,^47^,^48^. Studies demonstrated that c-Maf regulated the expression of IL-10 under Th1 and Th17 cell polarization condition^46^,^49^ and the enforced c-Maf expression induced IL-10 and IL-4 production in T cells. Several reports showed that IL-27 cooperated with TGF-β in activation of c-Maf and lead to the induction of IL-10-producing CD4^+^Foxp3^−^ Tr1 cells^19^,^20^,^21^,^27^,^50^,^51^,^52^. Adoptive transfer of B cells attenuated DSS-induced colitis in an IL-10-independent manner^11^ and B cells ameliorated T-cell-mediated inflammatory bowel disease by inducing Tr1 cells in an IL-27-depedent manner^10^. These findings are consistent with that previous results from our laboratory showed that IL-10-deficient B cells induced functional Treg-of-B cells and suggested IL-10 was not essential for the generation of Treg-of-B cells^29^. Indeed, the present report confirmed the dispensable role of IL-10 by IL-10-deficient B cell and neutralization of IL-10 during Treg-of-B3 cells generation. Neutralization of IL-10 and IL-27 did not alter the Treg-of-B3 cells induction, although the levels of IL-10, IL-27, and TGF-β increased during Treg-of-B3 cells induction. In conclusion, the antigen-presenting B cells induced IL-10-producing regulatory T cells might be through the activation of c-Maf by cell-cell contact.

We herein suggested that cell-cell contact was required for Treg-of-B cells generation while the transwell insertion reversed the suppressive function and expressions of IL-10, c-Maf, CTLA4, PD1, and antigen-specific TCR. Previous *in vitro* cell culture system demonstrated that splenic B cells required cell-cell contact including CD80 and CD86 costimulation for Treg-of-B cells induction^29^. In consistent with these, B-cell-induced T cell generated with additional anti-CD28 antibody decreased suppressive ability^53^. Some reports suggested that ICOS-ICOSL axis played a role in the induction of IL-10-producing Treg cells^24^,^25^,^43^,^54^, and ICOS regulated the suppressive function of Treg cells^43^,^55^,^56^. Other studies showed that PD1 played a major role in the down-regulation of TCR signaling^57^, and PD1-PD ligands involved in the induction of peripheral tolerance^58^,^59^,^60^. These suggested that several pathways might involve in the fine-tuned generation mechanism of Treg-of-B cells.

In conclusion, the present study showed that repeated stimulation of B cells increased the expressions of IL-10, c-Maf, ICOS, and CTLA4 of Treg-of-B cells in an IL-10 and IL-27-independent and cell-cell contact dependent manner. Furthermore, antigen-specific Treg-of-B cells exerted IL-10 and CTLA4-dependent suppressive functions in both antigen-specific and non-antigen-specific fashion. We expect our findings might further explore the feasibility of application of manipulated B cells for tolerance and anti-tumor responses.

## Methods

### Animals

Female BALB/c mice were purchased from the National Laboratory Animal Center. The transgenic T cell receptor of DO11.10 mice recognizes the OVA_323–339_ peptide presented by H-2^d^. All mice used were between 6–12 weeks of age and maintained in specific pathogen-free conditions at Laboratory Animal Center of College of Medicine at National Taiwan University. All animal experiments were approved by the Institutional Animal Care and Use Committee at College of Medicine, National Taiwan University (license number 20130341), and performed in accordance with the approved guidelines.

### Preparation of Treg-of-B cells

Splenic B220^+^ and CD4^+^CD25^−^ cells were purified by immunomagnetic selection using magnetic nanoparticles conjugated antibodies (anti-Mouse CD45RB/B220 Magnetic Particles, BD Biosciences, San Jose, CA, USA; EasySep Mouse CD4^+^ T cell isolation kit, STEMCELL, Canada). Positive or negative selections were performed to purify each cell population according to the manufacturer’s instructions. The purity of the cells was confirmed by flow cytometry and was at least 95%.

As showed in Fig. 1A, naïve DO11.10 CD4^+^CD25^−^ cells were cultured with OVA_323–339_ peptide-pulsed BALB/c B220^+^ cells at a ratio of 1:1. After 3 days, half of medium were replaced with fresh medium and supplemented recombinant IL-2 (final concentration to 100 U mL^−1^, PeproTech, USA). After another 4 days, the dead cells in the coculture system were removed by Ficoll-Paque PLUS (GE Healthcare, Buckinghamshire, UK) and then living cells were cocultured with OVA_323–339_ peptide-pulsed B cells. The culture protocol was repeated for twice. The Treg-of-B (ToB)-1, -2, and -3 cells were collected by depleting B220^+^ cells at day 3, 10, and 17.

### Preparation of Tr1 cells

Naïve DO11.10 CD4^+^CD25^−^ cells were stimulated with irradiated and OVA_323–339_ peptide-pulsed cells in the presence of recombinant IL-10 (100 U mL^−1^, PeproTech). After 3 days, half of medium were replaced with fresh medium and supplemented recombinant IL-2 (final concentration to 100 U mL^−1^). After another 4 days, the dead cells were removed by Ficoll and then living cells were stimulated again. Tr1 cells were stimulated with antibodies for 3 times and collected by removing dead cells.

### Cytokine analysis

The purified cells were seeded at 1 × 10^6^ cells mL^−1^ and were stimulated with irradiated and OVA_323–339_ peptide-pulsed cells for 72 hours. Cytokine levels were measured by ELISA kit (DuoSet ELISA Development kit, R&D, Minneapolis, MN, USA) according to the manufacturer’s instructions.

### Real-time PCR analysis

Total RNA was extracted from cells by using TRIzol (Invitrogen) and then reversely transcribed to cDNA by using Scientific RevertAid Reverse Transcriptase (Thermo, Waltham, MA) according to the manufacturer’s instructions. A total 200 ng of RNA. The gene expression was quantified by using an IQ^2^ SYBR green ROX mix (Bio-genesis, Taipei, Taiwan) and was determined by normalizing with the gene *gapdh*. Data acquisition was performed on a StepOne Real-Time PCR System (Applied Biosystems, Life Technologies, CA, USA). The primers for real-timer PCR were: *c-Maf*, AGC AGT TGG TGA CCA TGT GG and TGG AGA TCT CCT GCT TGA GG; *Il10*, GGT TGC CAA GCC TTA TCG G and TCT TCA CCT GCT CCA CTG C; *Il4*, TGT CAT CCT GCT CTT CTT TCT C and TCT GTG GTG TTC TTC GTT GC; *gapdh*, GAT GGG TGT GAA CCA CGA GA and AGA TCC ACG ACG GAC ACA T.

### Flow cytometry

Flow cytometry was performed according to standard procedures. Data acquisition was performed on a FACSCalibur (BD), and data were analyzed using the CellQuest Pro software (BD).

The following listed fluorescent-labeled anti-mouse monoclonal antibodies (and clone names) all purchased from eBioscience (San Diego, CA): B220 (RA3-6B2), CD4 (GK1.5), DO11.10 TCR (KJ1-26), CD25 (PC61.5), ICOS (C398.4 A), PD1 (J43), CTLA4 (UC10-4B9), Helios (22F6), Foxp3 (FJK-16s), LAG3 (C9B7W), c-Maf (sym0F1), IL-4 (11B11), IL-10 (JES5-16E3) and IFN-γ (XMG1.2).

### ^3^H incorporation assay

For the antigen-specific suppressive assay, 1 × 10^5^ DO11.10 splenic CD4^+^CD25^−^ T cells as responder cells were stimulated with γ-irradiated (3000 rad) and OVA_323–339_ peptide-pulsed cells. The suppressor cells were seeded with responder cells at a ratio of 1:1. [^3^H]-thymidine (1 μCi in each well, PerkinElmer, Boston, MA) was added after 3 days, and the cells were harvested after 16–18 hours. During the neutralization or blockade assay, cells were treated with the anti-CD16/32 antibody (101310, BioLegend, San Diego, CA) prior neutralizing antibody, including anti-IL-10 (554463, BD), anti-IL-10 receptor (55012, BD), anti-CTLA4 (106204, BioLegend), anti-LAG3 (552379, BD), and the relative isotype control antibodies. The results were presented in counts per minute (c.p.m.).

### Statistical analysis

Statistical analysis was performed with Mann-Whitney test or one-way ANOVA, where appropriate, by using GraphPad Prism v.5.0 Software (GraphPad, San Diego, CA). A *p*-value < 0.05 was considered as statistical significance. *p < .05; **, *p* < .01; ***p < .001. All data expressed as mean ± SD.

## Additional Information

**How to cite this article:** Chien, C.-H. *et al*. Characterization of c-Maf^+^Foxp3^-^ Regulatory T Cells Induced by Repeated Stimulation of Antigen-Presenting B Cells. *Sci. Rep.* **7**, 46348; doi: 10.1038/srep46348 (2017).

**Publisher's note:** Springer Nature remains neutral with regard to jurisdictional claims in published maps and institutional affiliations.

## Supplementary Material

Supplementary Data
