# Supplementary material for: Characterization of c-Maf+Foxp3− Regulatory T Cells Induced by Repeated Stimulation of Antigen-Presenting B Cells
Source: Sci Rep. 2017 Apr 12;7:46348. doi: 10.1038/srep46348 (PMC5389357; doi:10.1038/srep46348)
Supplement: Supplementary Data [file srep46348-s1.pdf]

Supplementary information

Manuscript: SREP-16-42480A

Characterization of c-Maf^{+}Foxp3^{-} Regulatory T Cells Induced by Repeated Stimulation of Antigen-Presenting B Cells

Chien-Hui Chien[1], Hui-Chieh Yu[1], Szu-Ying Chen[1], and Bor-Luen Chiang[1, 2,] *

[1] Graduate Institute of Clinical Medicine, National Taiwan University, Taipei City, 10048, Taiwan (R.O.C.)

[2] Department of Medical Reseach, National Taiwan University Hospital, Taipei City, 10002, Taiwan (R.O.C)

Methods

Animals

Female *Il10*$^{-/-}$ (BALB/c background) mice were purchased from Jackson Laboratory. All mice used were between 6-12 weeks of age and maintained in specific pathogen-free conditions at Laboratory Animal Center of College of Medicine at National Taiwan University. All animal experiments were approved by the Institutional Animal Care and Use Committee at College of Medicine, National Taiwan University (license number 20130341), and performed in accordance with the approved guidelines.

Neutralization in generation of Treg-of-B cells

B cells were treated with the anti-CD16/32 antibody (101310, BioLegend, San Diego, CA) prior neutralizing antibody, including anti-IL-10 (554463, BD), anti-IL-27 (516912, BioLegend), anti-TGF-β (141304, BioLegend), and their isotype control antibodies.

Preparation of non-antigen-specific Treg-of-B cells

Magnetic purified B220^{+} and CD4^{+}CD25^{-} T cells from naïve BALB/c mice were cocultured at a ratio of 1:1 in the presence of anti-CD3 (100331, BioLegend) and anti-CD28 (102112, BioLegend) monoclonal antibodies. After 3 days, Treg-of-B cells were collected by depleting B cells for further analysis.

Non-antigen-specific suppression assay

Female BALB/c mice were sensitized with β-lactoglobulin (BLG, L3908; Sigma-Aldrich, St Louis, MO) or human serum albumin (HSA, A9731; Sigma-Aldrich) emulsified with aluminum adjuvant (77161; Thermo Scientific, Waltham, Mass) for 4 times. To enrich BLG or HSA-specific T cells, splenic CD4^{+} T cells were purified after splenocytes stimulation with antigen and then resting for further experiments. As responder cell in the non-antigen-specific suppressive assay, enriched antigen-specific CD4^{+} T cells were stimulated with γ-irradiated, antigen-treated, and $OVA_{323-339}$ peptide-pulsed cells.

Figures

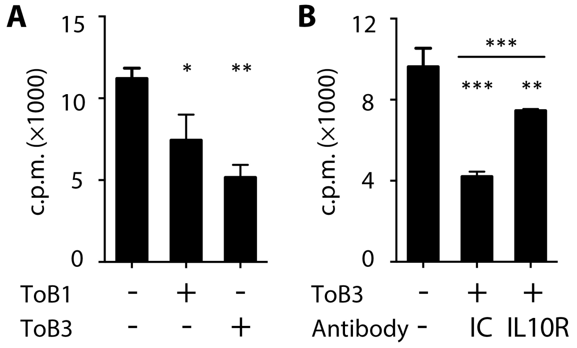

Supplementary Figure 1. Non-antigen-specific suppressive ability of Treg-of-B3 cells. **(A)** Non-antigen-specific suppressive function of ToB1 and ToB3 were determined by utilizing enriched human serum albumin (HSA)-specific $CD4^+$ T cell as responder cells. Data shown were the representative of 2 individual experiments. **(B)** Suppression assay was performed in the presence of 10 μg mL^{-1} anti-IL-10 receptor (IL10R) or isotype control (IC) antibodies and enriched β-lactoglobulin (BLG)-specific $CD4^+$ T cell as responder cells. Data shown were the representative of 2 individual experiments.

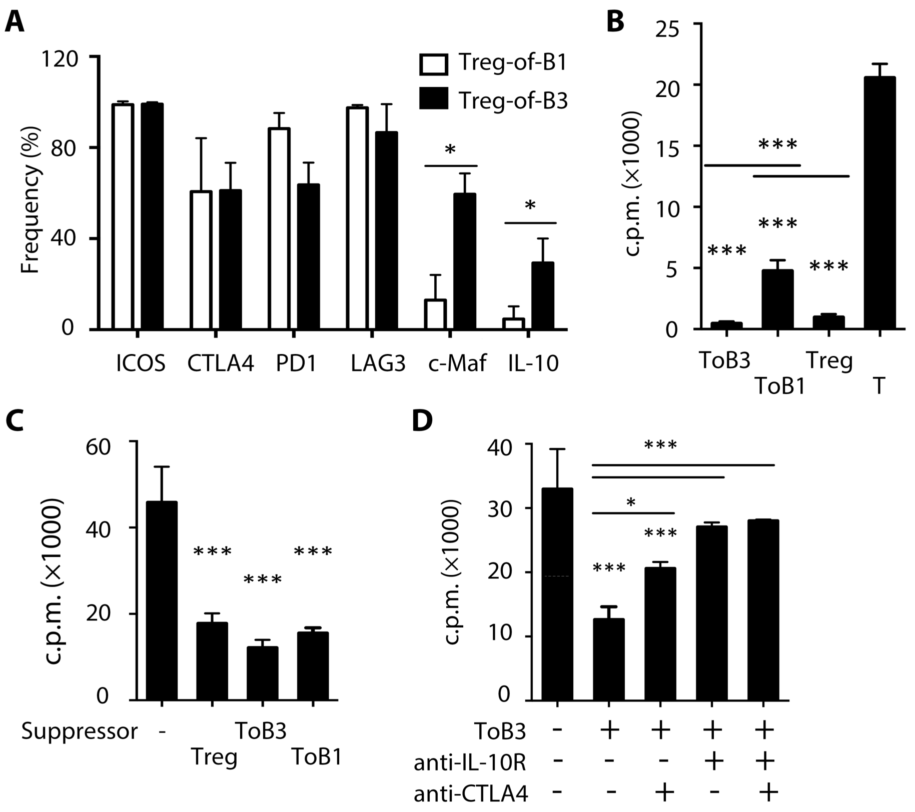

Supplementary Figure 2. Non-antigen-specific Treg-of-B3 cell shared characteristics of antigen-specific Treg-of-B3 cells. (A) The cumulative data of frequency of marker-expressing populations in anti-CD3 and anti-CD28 monoclonal antibodies-induced (mAb-) Treg-of-B (ToB)-1 and -3 cells from 4-5 individual experiments. **(B)** Hypoproliferative ability of mAb-ToB1 and ToB3 cells were determined in the stimulation of irradiated splenocytes and anti-CD3 and anti-CD28 monoclonal antibodies. Data shown were the representative of 2 individual experiments. **(C)** Suppressive function of mAb-ToB1 and ToB3 cells were determined, and BALB/c $CD4^{+}CD25^{-}$ T cells as responder cells stimulated with irradiated splenocyte in the presence of anti-CD3 and anti-CD28 monoclonal antibodies. Data shown were the representative of 2 individual experiments. (D) Suppression assay was performed in the presence of 10 μg mL^{-1} anti-IL-10 receptor (anti-IL-10R) and anti-CTLA4 antibodies or not. Data shown were the representative of 2 individual experiments.

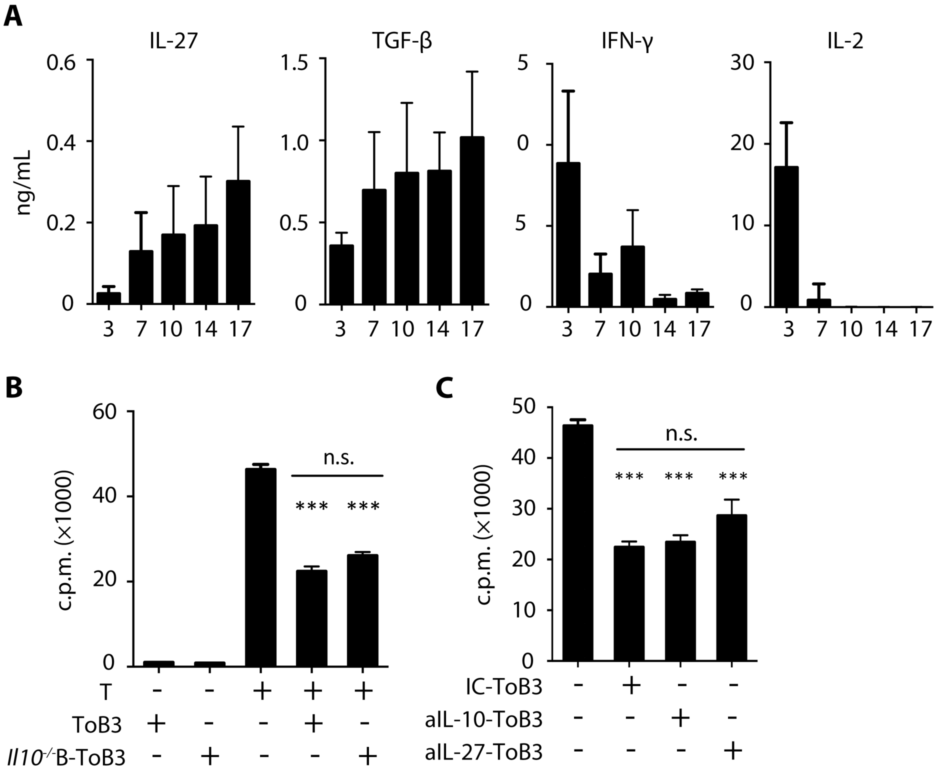

Supplementary Figure 3. Not IL-10 or IL-27 play the crucial role of Treg-of-B3 cell induction. (A) The cumulative data of cytokine levels of Treg-of-B3 cells during the culture period from 3 individual experiments. **(B)** Suppressive function of Treg-of-B3 cells induced by BALB/c B cells and IL-10-deficient B (*Il10*$^{-/-}$B-ToB3) cells were examined once. **(C)** Suppressive function of Treg-of-B3 cells induced with neutralizing antibodies against IL-10 or IL-27 and TGF-β were determined. Data shown were the representative of 3 individual experiments.

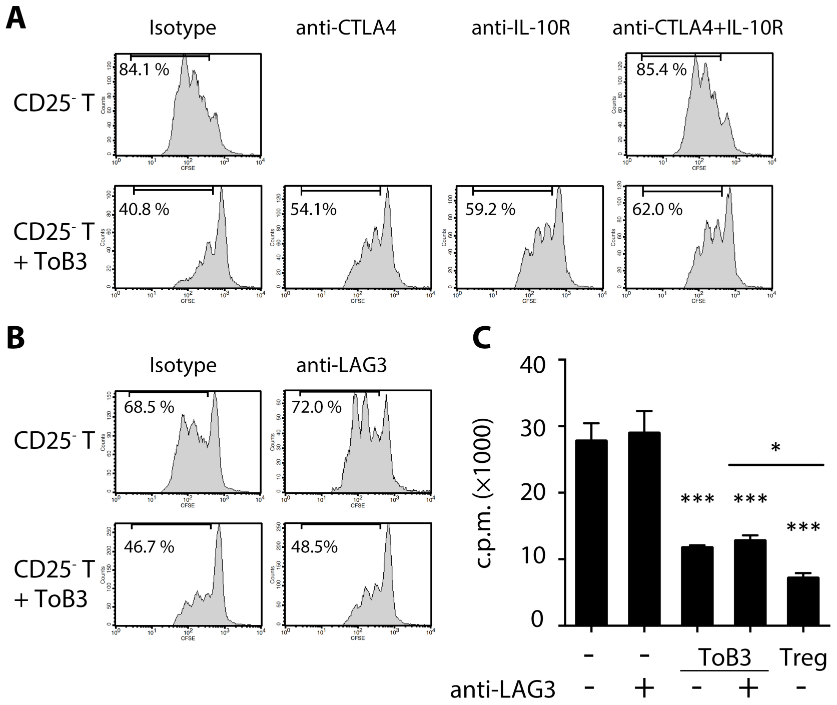

Supplementary Figure 4. Treg-of-B3 cells exerted CTLA4 and IL-10-dependent suppressive function. (A) DO11.10 CD25- T cells were labeled with carboxyfluorescein succinimidyl ester (CFSE) and stimulated with $OVA_{323\text{-}339}$ peptid-pulsed splenocytes for 3 days. The Treg-of-B3 (ToB3) cells seed at a ratio of 1:1 to the $CD25^-$ T cells in the presence of anti-CTLA4 and/or anti-IL-10 receptor or relative isotype antibodies. The frequency indicated the proliferated $CD25^-$ T cells. Data shown were the representative of 2 individual experiments. **(B)** CFSE-labeled CD25- T cells cultured with ToB3 cells in the presence of anti-LAG3 or isotype antibodies for 3 days. **(C)** Suppression assay was performed in the presence of anti-LAG3 or isotype antibodies.
